# Best Patient Care Practices for Administering PSMA-Targeted Radiopharmaceutical Therapy

**DOI:** 10.2967/jnumed.124.268363

**Published:** 2024-11

**Authors:** Jeremie Calais, Michael J. Morris, Ayse Tuba Kendi, Arash Rezazadeh Kalebasty, Ronald Tutrone, Michael J. Anderson, Oliver Sartor

**Affiliations:** 1 Ahmanson Translational Theranostics Division, Department of Molecular and Medical Pharmacology, UCLA, Los Angeles, California;; 2 Department of Genitourinary Oncology, Memorial Sloan Kettering Cancer Center, New York, New York;; 3 Department of Radiology, Mayo Clinic, Rochester, Minnesota;; 4 School of Medicine, University of California, Irvine, California;; 5 Chesapeake Urology Research Associates, Towson, Maryland;; 6 Department of Radiation Oncology, Comprehensive Cancer Centers of Nevada, Las Vegas, Nevada; and; 7 Departments of Urology and Oncology, Mayo Clinic, Rochester, Minnesota

**Keywords:** metastatic castration-resistant prostate cancer, prostate-specific membrane antigen, radiopharmaceutical therapy, multidisciplinary management, adverse events

## Abstract

Optimal patient management protocols for metastatic castration-resistant prostate cancer (mCRPC) are poorly defined and even further complexified with new therapy approvals, such as radiopharmaceuticals. The prostate-specific membrane antigen (PSMA)–targeted agent ^177^Lu vipivotide tetraxetan ([^177^Lu]Lu-PSMA-617), approved after the phase III VISION study, presents physicians with additional aspects of patient management, including specific adverse event (AE) monitoring and management, as well as radiation safety. Drawing on our experience as VISION study investigators, here we provide guidance on best practices for delivering PSMA-targeted radiopharmaceutical therapy (RPT) to patients with mCRPC. After a comprehensive review of published evidence and guidelines on RPT management in prostate cancer, we identified educational gaps in managing the radiation safety and AEs associated with [^177^Lu]Lu-PSMA-617. Our results showed that providing sufficient education on AEs (e.g., fatigue and dry mouth) and radiation safety principles is key to effective delivery and management of patient expectations. Patient counseling by health care professionals, across disciplines, is a cornerstone of optimal patient management during PSMA-targeted RPT. Multidisciplinary collaboration is crucial, and physicians must adhere to radiation safety protocols and counsel patients on radiation safety considerations. Treatment with [^177^Lu]Lu-PSMA-617 is generally well tolerated; however, additional interventions may be required, such as dosing modification, medications, or transfusions. Urinary incontinence can be challenging in the context of radiation safety. Multidisciplinary collaboration between medical oncologists and nuclear medicine teams ensures that patients are monitored and managed safely and efficiently. In clinical practice, the benefit-to-risk ratio should always be evaluated on a case-by-case basis.

Health care professionals (HCPs) have an increasing array of therapies to choose from when treating patients with metastatic castration-resistant prostate cancer (mCRPC) (1). Radiopharmaceutical therapy (RPT) is a novel treatment class in mCRPC; additional guidance on patient management is thus required.

The prostate-specific membrane antigen (PSMA)–targeted agent ^177^Lu-vipivotide tetraxetan ([^177^Lu]Lu-PSMA-617) is a Food and Drug Administration–approved RPT for mCRPC (2). In the prospective, open-label, randomized phase III VISION trial (NCT03511664), [^177^Lu]Lu-PSMA-617 plus investigator-chosen standard-of-care (SoC) therapy provided a significant survival benefit versus SoC alone in patients with previously treated PSMA-positive mCRPC (3). After these data were obtained, [^177^Lu]Lu-PSMA-617 was approved for the treatment of adults with previously treated PSMA-positive mCRPC (2)—a heavily pretreated population with comorbidities (3,4) that can further complicate management.

With the clinical integration of new therapies for mCRPC, advances in the treatment landscape have outpaced the ability of guidelines to provide universal recommendations (5). Consequently, HCPs require clear practical guidance on best practices for the effective and safe use of these new therapies. Notably, RPT (e.g., [^177^Lu]Lu-PSMA-617), in particular, presents HCPs with unique considerations for optimal delivery and patient management; seamless cross-disciplinary collaboration is required to optimize patient management (5,6) and ensure that RPT-specific considerations are addressed. This review draws on the experience of the VISION study investigators to provide guidance on best practices for delivering PSMA-targeted RPT, with a focus on adverse event (AE) management and radiation safety.

## BEST PRACTICES FOR AE MANAGEMENT

The safety of [^177^Lu]Lu-PSMA-617 in patients with mCRPC has been established in the phase III VISION study (3,7) and the phase II studies TheraP and RESIST-PC (8,9). In the VISION study, the most commonly reported AEs with [^177^Lu]Lu-PSMA-617 treatment were fatigue, dry mouth, nausea, and anemia (Supplemental Table 1; supplemental materials are available at http://jnm.snmjournals.org) (3), generally consistent with reports from TheraP (8) and RESIST-PC (9). Of note, a subgroup analysis of the VISION study noted that the incidence of treatment-emergent AEs decreased with [^177^Lu]Lu-PSMA-617 between the first 4 cycles and the next 2 cycles (5 and 6), reducing from 65% (*n* = 240) at cycle 4 to 57% (*n* = 170) at cycle 5 and 47% (*n* = 121) at cycle 6 (7).

Generally, AEs associated with [^177^Lu]Lu-PSMA-617 treatment can be managed according to the prescribing information (2) and guidance from procedure guidelines (10); dose reduction or permanent discontinuation of [^177^Lu]Lu-PSMA-617 may be needed in some cases (Supplemental Table 2). To further facilitate management, patients and caregivers should be clearly informed of the description, expected rates, and management strategies for AEs at the first consultation.

However, despite current methods, some AEs (e.g., dry mouth) remain a challenge to manage (11). Additional tools such as the Functional Assessment of Cancer Therapy–Radionuclide Therapy (FACT-RNT) have been developed to monitor relevant symptoms and toxicities among patients with prostate cancer (PC) in radiopharmaceutical trials and real-world settings (12), but there remains a lack of consensus on their management. Below we highlight common AEs associated with [^177^Lu]Lu-PSMA-617 and discuss potential management strategies.

### Fatigue

Fatigue was the most common AE in VISION, reported in 43.1% of [^177^Lu]Lu-PSMA-617–treated patients; few events were grade 3 or higher (5.9%). Of note, nearly a quarter of patients in the SoC-alone arm also reported fatigue in VISION (22.9%) (3). Although additional safety analyses of VISION revealed that the incidence of fatigue AEs tended to decline with subsequent cycles of [^177^Lu]Lu-PSMA-617 (from 24% during cycle 1 to 7.0% in cycle 6) (7), fatigue remains a common AE encountered by HCPs in patients with PC and is associated with a negative impact on patient quality of life (13).

Fatigue represents a complex issue that does not have one management consensus; available strategies that HCPs can use include pharmaceuticals (e.g., methylphenidate), transfusion (for anemia-related fatigue), psychologic support, or exercise treatments (e.g., aerobic and resistance exercises) (14–16). To facilitate adequate detection of this AE with RPT, manifestations of fatigue have been added to the FACT-RNT (12) and can be used in early discussions with patients.

### Dry Mouth

The salivary glands are among the organs with the highest absorbed doses of radiation after [^177^Lu]Lu-PSMA-617 administration (17) and therefore may be affected during treatment (11,18). Salivary toxicity is a common side effect of PSMA-targeted RPT, reported to be associated with higher treatment responses (19), and remains a management challenge (11). Low-grade (grade 1–2) dry mouth was a common AE in VISION, reported in 38.8% of [^177^Lu]Lu-PSMA-617–treated patients versus 0.5% of SoC-treated patients; no patients in either group experienced grade 3–4 events (3).

**NOTEWORTHY**

- As the treatment landscape in advanced PC evolves toward targeted treatments, HCPs must become familiar with novel therapies and associated management strategies.
- In this review, investigators from the VISION trial (a phase III clinical trial evaluating [^177^Lu]Lu-PSMA-617 in patients with mCRPC) provide expert guidance on best practices for delivering PSMA-targeted RPT in mCRPC.
- Close collaboration between HCPs across disciplines is vital to ensuring adherence to radiation safety and monitoring/management of AEs.

Patients should be assessed for symptoms of dry mouth both before initiating treatment with [^177^Lu]Lu-PSMA-617 and at subsequent follow-ups to determine its severity and to distinguish between nocturnal dryness and symptoms of dry mouth that limit oral function (20). Patients with mCRPC who present with preexisting dry mouth can be at greater risk of developing long-term complications (11) and should be monitored closely for worsening symptoms. Future studies are required to assess whether dry-mouth symptoms can be anticipated individually with PSMA PET (21). To ensure adequate detection of this AE in RPT, physicians can use the FACT-RNT (12).

Although there is no consensus on dry-mouth management, Muniz et al. recently provided an overview of the preventive and palliative measures that physicians can use (11). In VISION, patients experiencing dry mouth were advised to use sodium bicarbonate mouthwash during the first 3 d of each cycle (3). In clinical practice, additional strategies have been used, including good hydration and use of saliva gel to stimulate salivary flow; other strategies include parasympathomimetics (e.g., pilocarpine), hard sugarless candies or gum, xylitol, and gum disks (2,10,11,22). Of note, no controlled studies testing these strategies have been conducted.

### Hematologic Toxicity

After systemic administration of radionuclides, radiation from the blood and scattering from bone metastases may lead to myelosuppression (23). In VISION, the most common manifestation of myelosuppression was anemia; 31.8% and 12.9% of patients in the [^177^Lu]Lu-PSMA-617–plus–SoC arm experienced all-grade and grade 3–4 anemia, respectively, versus 13.2% and 4.9% in the SoC-alone arm (3).

Candidates for treatment with [^177^Lu]Lu-PSMA-617 can present with preexisting myelosuppression due to previous treatment with chemotherapy regimens or poly-ADP ribose polymerase inhibitors (23–25). In addition, patients who have recently undergone a bone marrow transplantation may also be considered for treatment with [^177^Lu]Lu-PSMA-617 (20). As it is not recommended to wait for function recovery before initiating treatment, patients may start treatment while presenting with a rapidly progressing bone marrow dysfunction.

In addition, patients with advanced PC frequently present with preexisting anemia due to multiple factors, including androgen deprivation, nutritional decline, bone marrow infiltration, and treatment-related toxicity (15). Such patients may require medical intervention to manage hematologic toxicities during treatment with [^177^Lu]Lu-PSMA-617 (26).

Low blood cell counts (anemia, thrombocytopenia, and leukopenia [including neutropenia]) were the most common cause of dose reduction, interruption, and permanent discontinuation of [^177^Lu]Lu-PSMA-617 in the phase III VISION study (Supplemental Table 2 provides dose modification recommendations) (2). Signs of myelosuppression and anemia should be monitored with complete blood counts before and during treatment with [^177^Lu]Lu-PSMA-617 (2). Further interventions may be required to resolve hematologic toxicities after consulting with a hematologist (10,20).

Collaboration between HCPs and radiologists or nuclear medicine physicians can be particularly beneficial in anticipating and managing hematologic toxicities, as the presence and extent of the bone disease can be visualized by PSMA PET and CT scans (23,27). This was further highlighted by recent studies noting that PSMA PET bone tumor volume is associated with hematologic toxicity, after adjustment for other clinical variables (28,29). Despite this association, [^177^Lu]Lu-PSMA-617 remains efficacious in patients with diffuse bone involvement (30).

### Gastrointestinal Toxicity

In VISION, gastrointestinal toxicities (all grades) in the [^177^Lu]Lu-PSMA-617–plus–SoC arm included nausea (35.3%), constipation (20.2%), vomiting (18.9%), diarrhea (18.9%), and abdominal pain (6.0%) (3). Although not required per prescribing information, VISION participants were managed using prophylactic antiemetics, if needed (3). It is recommended to pause treatment with [^177^Lu]Lu-PSMA-617 in patients who experience toxicities of grade 3 or higher, including gastrointestinal toxicities, until a grade of 2 or less is reached (10).

To ensure adequate detection of gastric AEs during RPT treatment, physicians can report several manifestations of gastrointestinal toxicity (e.g., nausea, vomiting, diarrhea, and constipation) using the FACT-RNT (12).

### Dry Eye

In VISION, dry eye was reported in 3.0% of patients in the [^177^Lu]Lu-PSMA-617–plus–SoC arm (all grade 1–2) (3). Although its reported incidence was relatively low in VISION, dry eye has also been reported with [^177^Lu]Lu-PSMA-617 in other studies, such as RESIST-PC (6.3%, all nonsevere) (9) and TheraP (30%, all grade 1–2) (8). The lacrimal glands are exposed to relatively high doses of radiation after [^177^Lu]Lu-PSMA-617 administration (31); thus, dry eye is considered a clinically relevant symptom in PSMA-targeted RPT and has been reported to be associated with higher treatment responses (19). Ophthalmologic AEs occurring in patients treated for PC can be underreported (32). To facilitate adequate detection of this AE, physicians can use FACT-RNT (12). Generally, there is no consensus on dry-eye management, but physicians can delay the next cycle of [^177^Lu]Lu-PSMA-617 until resolution of symptoms (33). In clinical practice, use of topical therapy with artificial tears, neomycin-polymyxin-dexamethasone suspensions, lubricating ointments, lid scrubs, and oral antihistamines has been reported to manage symptoms (34). Despite their association with dryness and discomfort, there is evidence that contact lenses may have a role in dry-eye management, and therapeutic soft contact lenses can be prescribed (34); however, patients may prefer to revert to glasses.

### Disease-Related AEs

Patients with mCRPC generally present with significant comorbidities and disease-related complications, including urinary symptoms, fatigue, bone pain or stiffness, or skeletal events (4,35,36). It is recommended to pause treatment with [^177^Lu]Lu-PSMA-617 until all nonhematologic AEs of grade 3 or higher resolve to grade 2 or less or as deemed appropriate by the treating physician (10).

Urinary comorbidities, such as urinary incontinence, are common among patients who are candidates for treatment with [^177^Lu]Lu-PSMA-617 (37). Although urinary incontinence and the use of a catheter do not represent a contraindication to treatment with [^177^Lu]Lu-PSMA-617 (10), they can have significant implications for radiation safety practices. Acute urinary tract obstruction and hydronephrosis should both be considered contraindications to treatment with [^177^Lu]Lu-PSMA-617. Patients with a history or risk of urinary retention should be assessed using renal scintigraphy at baseline to assess suitability for treatment (10).

Skeleton-related events have a negative impact on patients’ quality of life (38). In VISION, the time to the first symptomatic skeletal event (or death) was longer with [^177^Lu]Lu-PSMA-617–plus–SoC than with SoC alone (3); however, bone pain, which is associated with an increased risk of skeleton-related events (39), occurred in 11.2% (all grades) and 2.5% (grade 3–4) of patients receiving [^177^Lu]Lu-PSMA-617 (3). HCPs, through multidisciplinary collaboration, should learn how to manage these events and how to counsel patients to alleviate any anxieties.

Bone flares also occur in some patients with mCRPC (40), characterized by a transient increase in pain during the first week after [^177^Lu]Lu-PSMA-617 administration, which subsequently resolves (18). Antiinflammatory drugs such as dexamethasone can help decrease edema and pain (41). In severe cases, opioids can temporarily be used; however, an attempt should be made to reduce all treatments used to relieve symptoms of a bone flare, including analgesics, once interim laboratory tests demonstrate a prostate-specific antigen response (10). Collaboration of HCPs with radiologists and nuclear medicine physicians may help anticipate pain flares.

Neurologic complications may also develop during advanced PC, including spinal-cord compression caused by metastases, and brain metastases (the latter is rarer and represents disseminated disease). In both cases, treatment with dexamethasone as early as possible is recommended to decrease inflammation (42).

## BEST PRACTICES FOR RADIATION SAFETY IN PSMA-TARGETED AGENTS

For patients receiving [^177^Lu]Lu-PSMA-617, counseling on radiation safety precautions by HCPs is essential; because of their close day-to-day contact with patients (43), nuclear medicine physicians and nuclear medicine nurses can be ideal candidates. Radiation safety discussions provide an opportunity to dispel any patient misconceptions regarding PSMA-targeted RPT. [^177^Lu]Lu-PSMA-617 has a physical half-life of 6.65 d and decays to a stable state by emitting β^−^ and γ-radiation (2). In the days after [^177^Lu]Lu-PSMA-617 administration, patients and their bodily fluids will continue to be a source of radiation (43). To provide context on the levels of radiation exposure with radiopharmaceuticals, HCPs should communicate that these levels are comparable to those of diagnostic x-ray imaging (44,45), a procedure with which most patients are familiar. To improve confidence and provide assurance, key safety measures, rules on transportation, and other logistic considerations for radiopharmaceuticals should be explained to patients and their caregivers (Supplemental Table 3) and outlined on printed information sheets (Supplemental Fig. 1) (46,47).

A radiation safety card detailing the treatment and radioactivity amount should be given to patients after each cycle of therapy, to be carried at all times during and 3 mo after the final cycle of treatment (46). This card should be presented to security personnel when traveling or to medical staff when receiving treatment. In some cases, patients may be required to travel in the days and weeks after the administration of [^177^Lu]Lu-PSMA-617 and thus should receive advice. As stated in Supplemental Table 3, patients should refrain from traveling close to others for 3 d after the administration of [^177^Lu]Lu-PSMA-617. Radioactivity from [^177^Lu]Lu persists at low levels in patients for several weeks after treatment (48) and is not harmful but can be detected by sensitive radiation detectors at international airports or border crossings (46). This can cause delays due to additional screening procedures; HCPs should advise patients to present their radiation safety card and a copy of their most recent clinic notes to security personnel if stopped (46).

As patients with mCRPC can often present with comorbidities (4), urgent care may be required after treatment. Patients should not be discouraged from seeking urgent medical attention but should be advised, along with their caregivers, to present their radiation safety card to the clinical care team and inform the team of the relevant radiation safety instructions (18,49).

As most [^177^Lu]Lu-PSMA-617 is excreted via urine (2,50), urinary incontinence represents one of the most significant logistic challenges for HCPs administering [^177^Lu]Lu-PSMA-617. This is compounded by the increased prevalence of urinary incontinence among patients with metastatic PC (37). Incontinence pads should be used and frequently changed during radiopharmaceutical administration—for example, [^177^Lu]Lu-PSMA-617—to reduce radiation exposure and contamination and to avoid radiation burns from urinary leakages (18,51). Disposal precautions similar to those used for other radiopharmaceuticals can also be used by patients (52); during the first week after [^177^Lu]Lu-PSMA-617 administration, any items that cannot be flushed down the toilet, such as sanitary pads and bandages, must be placed in specific plastic trash bags and can be thrown away with other household waste after 70 d. The care team involved in the administration of [^177^Lu]Lu-PSMA-617, particularly nursing professionals, should be aware of the type of nephrostomy bags required and should obtain additional bags to facilitate frequent changing (18); individuals in the care facility setting should also have an appreciation of any patient requirements. A Foley catheter with acrylic shielding of the Foley bag may be required for patients with urinary incontinence (51) during the administration of PSMA-targeted RPT. In certain cases, particularly if the patient experiencing incontinence lives with children, hospitalization may be required to avoid unnecessary exposure even when the external dose rate is sufficiently low (49).

All personnel involved in the administration of PSMA-targeted RPT may be exposed to an increased amount of γ- and β-radiation (2). An individual’s cumulative exposure to radiation is associated with a potential increased risk for cancer (2). Therefore, all personnel involved in the administration of PSMA-targeted RPT must follow institutional good-radiation-safety practices and patient treatment procedures (2). As guidance can differ between institutions, HCPs must feel equipped to facilitate safe administration of PSMA-targeted RPT and adapt institutional guidelines to accommodate individual patient characteristics.

The safe disposal of waste materials is an essential aspect of ensuring radiation safety. It is imperative that any unused medicinal products or waste materials be disposed of in accordance with local and federal laws (2). An accredited radiation safety officer within an institution should be appointed to manage these additional considerations during the handling and decay of any radioactive waste (53), including disposal of residual or unused doses and contaminated materials (43). The radiation safety officer should also provide training to nuclear medicine staff (including nuclear medicine nurses and technologists) (43), as well as to professionals in care facility settings.

In the case of patient death after treatment with [^177^Lu]Lu-PSMA-617, care teams should be informed of the potential radioactivity of the deceased. Access to the room occupied by the deceased should be restricted until decontaminated and appropriately surveyed, and radioactive corpses should be clearly labeled as potentially hazardous and kept in body bags in case of liquid leakage. Appropriate surveillance may be required through the disposal process, and a radiation protection officer should be called to supervise the handling of a significantly radioactive corpse (49). The death certificates of deceased patients should be appropriately labeled (51). Radiation safety officers should appropriately train the medical examiners and mortuary personnel, as well as perform radiation surveys (49,51).

## BEST PRACTICES FOR MULTIDISCIPLINARY MANAGEMENT OF MCRPC DURING PSMA-TARGETED RPT

Multidisciplinary collaboration between treating HCPs and nuclear medicine teams is crucial to the management of patients receiving PSMA-targeted RPT (6,54).

During patient selection for treatment with [^177^Lu]Lu-PSMA-617, HCPs should consider clinical characteristics and the results of any imaging tests to determine the optimal treatment strategy, because patients may also be eligible for other treatments (e.g., cabazitaxel) (55). Multidisciplinary consultation can be particularly valuable in unclear or borderline cases, for which insight from various disciplines may be required to make treatment decisions, such as when treatment discussions involve a variety of imaging modalities or histopathologic confirmation (5).

Multidisciplinary expertise helps in the management of disease- and treatment-related AEs occurring in this heavily pretreated and advanced-disease population. During [^177^Lu]Lu-PSMA-617 treatment, it is particularly important that cross-functional communication occur after the fourth dose to determine whether a patient will proceed with all 6 doses (53) or whether treatment should be paused or discontinued to manage any AEs. Treatment optimization can be facilitated by incorporating dosimetry scan data (56). Some centers may also acquire SPECT images of the [^177^Lu]Lu-PSMA-617 therapy up to 24 h after administration of each cycle (57) for therapy response assessment. These assessments can lead to further therapy adjustment, such as reducing the frequency of injections and increasing the time interval between injections.

Another scenario that highlights the importance of multidisciplinary communication is treatment delay if the patient develops a contraindication to [^177^Lu]Lu-PSMA 617 treatment between screening and the first administration (10).

Patient counseling by HCPs, across disciplines, is a cornerstone of optimal patient management during PSMA-targeted RPT. It is of particular importance when monitoring patients for treatment-related AEs, as some AEs may be underreported (58) if patients are not asked directly about their incidence (e.g., areas of patient sensitivity such as treatment-emergent sexual dysfunction) (59), and lack of early intervention to resolve emergent AEs may result in their worsening and greater clinical sequelae. HCPs, including nuclear medicine nurses, should establish open dialogues with patients and ask open-ended questions (18,43), such as by providing patients with a designated hotline or telephone number that they can call to report any new signs or symptoms of AEs. These approaches allow more detailed feedback to be received from patients during routine examinations and increase the likelihood of identifying potential AEs (18).

Physicians may refer to the standard operating procedure published by Calais et al. for additional guidance on the incorporation of RPT in clinical practice, including details on actions to perform at screening and throughout treatment cycles, roles and responsibilities, and pertinent documentation for HCPs (e.g., injection methods) and patients (e.g., FACT-RNT sheet, discharge instructions) (43).

## CONCLUSION

When treating patients with mCRPC using PSMA-targeted RPTs, HCPs may encounter a range of new challenges. Effective decision-making in these circumstances necessitates collaboration across various medical disciplines.

Patient outcomes can be optimized when HCPs, across disciplines, are familiar with common disease- and treatment-related AEs in these patients and are aligned on radiation safety, including differences from other radiation-based treatments and precautions for health care personnel, patients, and caregivers. Adaptations to best practices may be required in community or rural settings, where availability of nuclear medicine expertise or specialized equipment (e.g., PSMA PET imaging) might be limited.

## DISCLOSURE

Jeremie Calais has received honoraria from RadioMedix Inc., Progenics Pharmaceuticals, Inc., Advanced Accelerator Applications, and EXINI Diagnostics AB; has received research funding from Progenics Pharmaceuticals, Inc.; acts in a consulting or advisory role for Blue Earth Diagnostics, Janssen Pharmaceuticals, Progenics Pharmaceuticals, Inc., Curium Pharma, GE HealthCare, Telix Pharmaceuticals, POINT Biopharma, and Lantheus Medical Imaging; and has participated in speakers’ bureaus for Telix Pharmaceuticals and IBA RadioPharma Solutions. Michael Morris has consulting or advisory arrangements with Bayer, Endocyte, Advanced Accelerator Applications, ORIC Pharmaceuticals, Johnson & Johnson, Curium Pharma, and Athenex; has received paid travel from Endocyte and Fujifilm; and has received research funding from Bayer, Sanofi, Endocyte, Progenics Pharmaceuticals, Inc., Corcept Therapeutics, Roche/Genentech, and Janssen Pharmaceuticals. He has a patent pending with Novartis and receives royalties from Telix. Ayse Tuba Kendi has participated in an advisory board regarding Lu-PSMA research, funded by Novartis. Arash Rezazadeh Kalebasty has stock and other ownership interests in ECOM Medical; has acted in a consulting or advisory role for Exelixis, AstraZeneca, Bayer, Pfizer, Novartis, Genentech, Bristol Myers Squibb, EMD Serono, Immunomedics, and Gilead Sciences; has participated in speakers’ bureaus for Janssen, Astellas Medivation, Pfizer, Novartis, Sanofi, Genentech/Roche, Eisai, AstraZeneca, Bristol Myers Squibb, Amgen, Exelixis, EMD Serono, Merck, Seattle Genetics/Astellas, Myovant Sciences, Gilead Sciences, and AVEO Oncology; has received research funding from Genentech, Exelixis, Janssen, AstraZeneca, Bayer, Bristol Myers Squibb, Eisai, Macrogenics, Astellas Pharma, BeyondSpring Pharmaceuticals, BioClin Therapeutics, Clovis Oncology, Bavarian Nordic, Seattle Genetics, Immunomedics, Epizyme, Novartis, Arvinas, Amgen, POINT Biopharma, Merck, and Mirati Therapeutics; and has received travel accommodations and expenses from Genentech, Prometheus, Astellas Medivation, Janssen, Eisai, Bayer, Pfizer, Novartis, Exelixis, and AstraZeneca. Ronald Tutrone is a consultant/speaker for Astellas, Pfizer, Myovant, Dendreon, Bayer, Janssen, and Nymox and owns stock in Nymox, Veru, and Myovant. Michael Anderson has participated in a speakers’ bureau for Bayer. Oliver Sartor has received grants or contracts from Advanced Accelerator Applications, Amgen, AstraZeneca, Bayer, Constellation, Endocyte, Invitae, Janssen Pharmaceuticals, Lantheus, Merck, Progenics Pharmaceuticals, Inc., and Teneobio; has received consulting fees from Advanced Accelerator Applications, Astellas, AstraZeneca, Bayer, Blue Earth Diagnostics, Bavarian Nordic, Bristol Myers Squibb, Clarity Pharmaceuticals, Clovis, Constellation, Dendreon, EMD Serono, Fusion, Isotopen Technologien Meunchen, Janssen Pharmaceuticals, Myovant, Myriad, Noria Therapeutics, Novartis, Noxopharm, Progenics Pharmaceuticals, Inc., POINT Biopharma, Pfizer, Sanofi, Teneobio, Telix, and Theragnostics; has participated on data safety monitoring boards or advisory boards for AstraZeneca, Janssen, Pfizer, and Myovant; and holds stock or stock options in Clarity Pharmaceuticals, Noria Therapeutics, Eli Lilly, Clovis, GlaxoSmithKline, AbbVie, Cardinal Health, and United Health Group. Medical writing and editing assistance was provided by Pablo Izquierdo, PhD, and Sam Ffrench-Mullen, BSc, of Spark (a division of Prime, New York), funded by Novartis Pharmaceuticals Corp. All authors were principal investigators or investigators in the VISION study, sponsored by Novartis Pharmaceuticals Corp. This review was funded by Novartis Pharmaceuticals Corp. This article was developed in accordance with Good Publication Practice (GPP) guidelines. The authors had full control of the content and made the final decision on all aspects of this review. Neither Novartis Pharmaceuticals Corp. nor Prime influenced the content of this article, nor did the authors receive financial compensation for authorship. No other potential conflict of interest relevant to this article was reported.
